# Effect of Levothyroxine Therapy on the Lipid Profile of Patients With Hypothyroidism: A Systematic Review

**DOI:** 10.7759/cureus.65218

**Published:** 2024-07-23

**Authors:** Samreen Nishat, Isaac N Mueka, Maria U Hassan, Ravi K Pandey, Bo B Lwin, Apoorva Vashishta, Sondos T Nassar

**Affiliations:** 1 Clinical Research, California Institute of Behavioral Neurosciences & Psychology, Fairfield, USA; 2 Clinical Research, NewYork-Presbyterian Queens, New York, USA; 3 Medicine and Surgery, Jordan University of Science and Technology, Ar-Ramtha, JOR

**Keywords:** hypothyroidism, lipid panel, lipid profile, levothyroxine, dyslipidemia, low-density lipoproteins, cholesterol, triglycerides, thyroid hormone, cardiovascular risk

## Abstract

Hypothyroidism, also known as underactive thyroid, is a condition where the thyroid gland does not produce enough thyroid hormone. Deficiency or lack of thyroid hormone causes patients to have a slower metabolism, which may lead to secondary medical issues such as weight gain, fatigue, depression, and increased cardiovascular risk. This systematic review aims to explore the effect of levothyroxine therapy on the lipid profile of hypothyroid patients. Through a comprehensive search, 3096 articles were retrieved using keywords such as Hypothyroidism, Levothyroxine, Lipid, Dyslipidemia, and Cholesterol from PubMed, PubMed Central, Google Scholar, and ScienceDirect databases. The Medical Subject Headings (MeSH) strategy was also leveraged to extensively search the PubMed database. Research articles that were published from the year 2020 until May 2024, including randomized control trials, observational studies, meta-analyses, systematic reviews, literature reviews, and case reports, were included in the research. Research papers published before 2020, written in languages other than English, and animal studies were excluded. The 2020 Preferred Reporting Items for Systematic Reviews and Meta-Analyses (PRISMA) criteria were used in the design of the systematic review. Levothyroxine therapy is the treatment of choice in patients suffering from hypothyroidism, and based on our review, the treatment has a positive impact, leading to a significant decrease in total cholesterol, low-density lipoproteins, and triglyceride values in hypothyroid patients. The research highlights the importance of starting timely levothyroxine therapy in hypothyroid patients to maintain normal lipid levels and reduce the associated cardiovascular risk.

## Introduction and background

**Figure 1:**
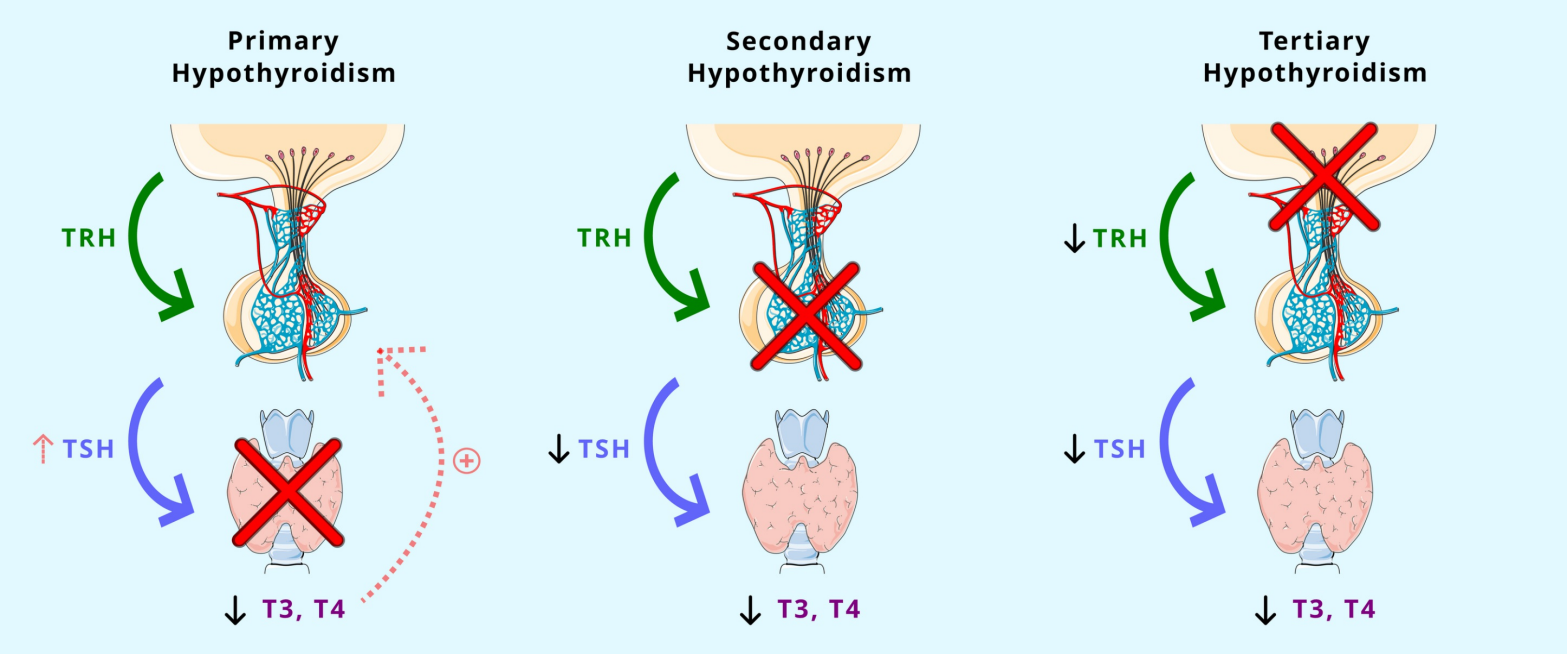
Mechanisms of the different types of hypothyroidism Primary hypothyroidism: decreased T3/T4 by the thyroid gland leads to a compensatory increase in TSH production; secondary hypothyroidism: decreased TSH due to pituitary disorders leads to decreased T3/T4; tertiary hypothyroidism: decreased TRH due to hypothalamic disorders leads to decreased TSH and T3/T4 TRH: thyrotropin-releasing hormone; T4: thyroxine; T3: triiodothyronine; TSH: thyroid-stimulating hormone Portions of the figure utilize assets from Servier Medical Art by Servier (https://smart.servier.com), licensed under a Creative Commons Attribution 4.0 International License (https://creativecommons.org/licenses/by/4.0)

Hypothyroidism is an endocrine disorder caused by the failure of the thyroid gland to produce adequate thyroid hormone, which affects the body's metabolism and may lead to obesity, fatigue, anorexia, hypertension, and hyperlipidemia [1]. It is divided into primary, secondary, and tertiary types. In primary hypothyroidism (PHT), the thyroid gland does not produce enough thyroid hormone, which results in a compensatory increase in thyroid-stimulating hormone (TSH). Secondary hypothyroidism is caused by pituitary disorders and is characterized by decreased levels of TSH and triiodothyronine (T3) or thyroxine (T4). Tertiary hypothyroidism is caused by hypothalamic dysfunction, which results in decreased levels of thyrotropin-releasing hormone (TRH), causing a decrease in TSH and T3/T4 [2-4]. Overt hypothyroidism is defined as elevated TSH and decreased thyroxine concentrations, whereas subclinical hypothyroidism (SCH) is defined as elevated TSH and a normal serum free thyroxine (FT4) level [5,6]. Both overt and SCH are associated with dyslipidemia [7]. Figure 1 illustrates the mechanisms of the different types of hypothyroidism.

Hypothyroidism affects up to 5% of the general population worldwide, with a further estimated 5% being undiagnosed. The National Health and Nutrition Examination Survey (NHANESIII) found the prevalence of overt hypothyroidism among individuals aged 12 years and above in the US to be 0.3% and subclinical hypothyroidism 4.3%. Iodine deficiency is the most common cause of hypothyroidism, but in areas of iodine sufficiency, Hashimoto’s disease is the most common cause of thyroid failure [2,5].

Thyroid dysfunction may alter the synthesis and degradation of lipids as well as the function of various enzymes in the lipid metabolism pathway. Insufficient production of thyroid hormones causes changes in the lipid profile, including levels of total cholesterol (TC), low-density lipoprotein (LDL), high-density lipoprotein (HDL), triglycerides (TG), and the levels of apolipoprotein A (apoA) and apolipoprotein B (apoB) [7-9]. In hypothyroidism, dyslipidemia is mainly caused by an increased synthesis rate compared to the degradation rate, resulting in elevated levels of TC, particularly LDL. These elevated lipid levels serve as substrates for lipid peroxidation by reactive oxygen species (ROS), resulting in oxidative stress [10]. The coexistence of dyslipidemia and hypothyroidism has emerged as a significant risk factor for atherosclerosis development. This combination can adversely affect cardiovascular disease risk by altering atherogenic lipid components, increasing arterial hypertension, and promoting inflammation and oxidative stress. These changes lead to endothelial dysfunction, which is the primary cause of death from coronary heart disease [11,12].

Levothyroxine (LT4) is the standard replacement therapy for hypothyroidism and is associated, through metabolic correction, with a substantial reduction in body weight and serum lipids [13]. The antioxidant and hypolipidemic properties of LT4 are proven, and LT4 therapy has a favorable effect on cardiovascular function and lipid profile [14]. The purpose of our study was to gather, evaluate, and present recent research and findings regarding the effect of LT4 treatment on the lipid profile of hypothyroidism patients.

## Review

Methodology

The 2020 updated Preferred Reporting Items for Systematic Review and Meta-Analyses (PRISMA) guidelines have been followed for the design and reporting of this systematic review [15].

Eligibility Criteria

The review question was formulated based on the population, intervention, and outcome (PIO) elements: the population includes patients diagnosed with hypothyroidism, the intervention is treatment with LT4, and the outcome includes any variation in the lipid profile of the patient. Besides these PIO elements, additional inclusion and exclusion criteria were used to narrow down the articles under consideration. The inclusion criteria included research articles with free full text in English published between 2020 and 2024, and human studies were included. The exclusion criteria were articles published before the year 2020, written in languages other than English, and animal studies.

Database and Search Strategy

Research articles from 2020 onwards until May 2024 were searched using the databases PubMed, PubMed Central, Google Scholar, and ScienceDirect using keywords and Medical Subject Headings (MeSH). All the research articles were imported to EndNote (Clarivate, Philadelphia, Pennsylvania), duplicates were removed, and the remaining research articles were screened manually by going through the titles and abstracts. After narrowing down and retrieving the most relevant papers, they were further analyzed by going through the full text. Quality assessment tools were used to remove the possibility of bias in this study. Table 1 includes the keywords and MeSH terms used in the databases.

**Table 1:** Details of the search strategy MeSH: Medical Subject Headings

Database	Keywords	Search Strategy	Filters	Search Result
PubMed	Hypothyroidism, subclinical hypothyroidism, levothyroxine, thyroxine, levo-thyroxine, lipid, dyslipidemia, cholesterol	(Hypothyroidism OR subclinical hypothyroidism OR ("Hypothyroidism/complications"(Mesh) OR "Hypothyroidism/drug therapy"(MeSH) OR "Hypothyroidism/metabolism"(MeSH) OR "Hypothyroidism/physiopathology"(MeSH) OR "Hypothyroidism/therapy"(MeSH))) AND (Levothyroxine OR thyroxine OR levo-thyroxine OR ("Thyroxine/adverse effects"(MeSH) OR "Thyroxine/drug effects"(MeSH) OR "Thyroxine/pharmacokinetics"(MeSH) OR "Thyroxine/pharmacology"(MeSH) OR "Thyroxine/physiology"(MeSH) OR "Thyroxine/therapeutic use"(MeSH))) AND (Lipid OR dyslipidemia OR Cholesterol OR ("Lipids/analysis"(MeSH) OR "Lipids/biosynthesis"(MeSH) OR "Lipids/blood"(MeSH) OR "Lipids/chemical synthesis"(MeSH) OR "Lipids/chemistry"(MeSH) OR "Lipids/physiology"(MeSH))) 1724 results without filters	2020-2024, free full text, English	138 results
Google Scholar	Hypothyroidism, levothyroxine, lipid profile	"Hypothyroidism" AND "Levothyroxine" AND "Lipid profile" 4,400 results without filters	2020-2024	1340 results
PubMed Central	Hypothyroidism, levothyroxine, lipid profile	Hypothyroidism AND Levothyroxine AND Lipid profile 3140 results without filters	2020-2024	1731 results
ScienceDirect	Hypothyroidism, levothyroxine, lipid profile	Hypothyroidism AND Levothyroxine AND Lipid profile 829 results without filters	2020-2024, Article type: review articles, research articles, case reports Access type: open access & ppen archive	51 results

Quality Assessment

A total of 15 selected studies were assessed for quality using standardized tools. The following tools were used for quality assessment: (1) Newcastle-Ottawa Scale (NOS) for cohort and case-control studies; (2) Assessment of Multiple Systematic Reviews 2 (AMSTAR 2) tool for systematic reviews and meta-analyses; and (3) Quality Assessment Tool for Before-After (Pre-Post) Studies with No Control Group by the National Institute of Health (NIH) for Pre-Post interventional studies [16-18].

Data Collection Process

Based on the results of the quality assessment tools, the research papers were shortlisted, and a comprehensive review of the shortlisted papers was conducted. Variation in the lipid profile values, which was the core outcome of the study, was evaluated. Most of the results showed how the lipid profile values changed after LT4 administration.

Results

**Figure 2:**
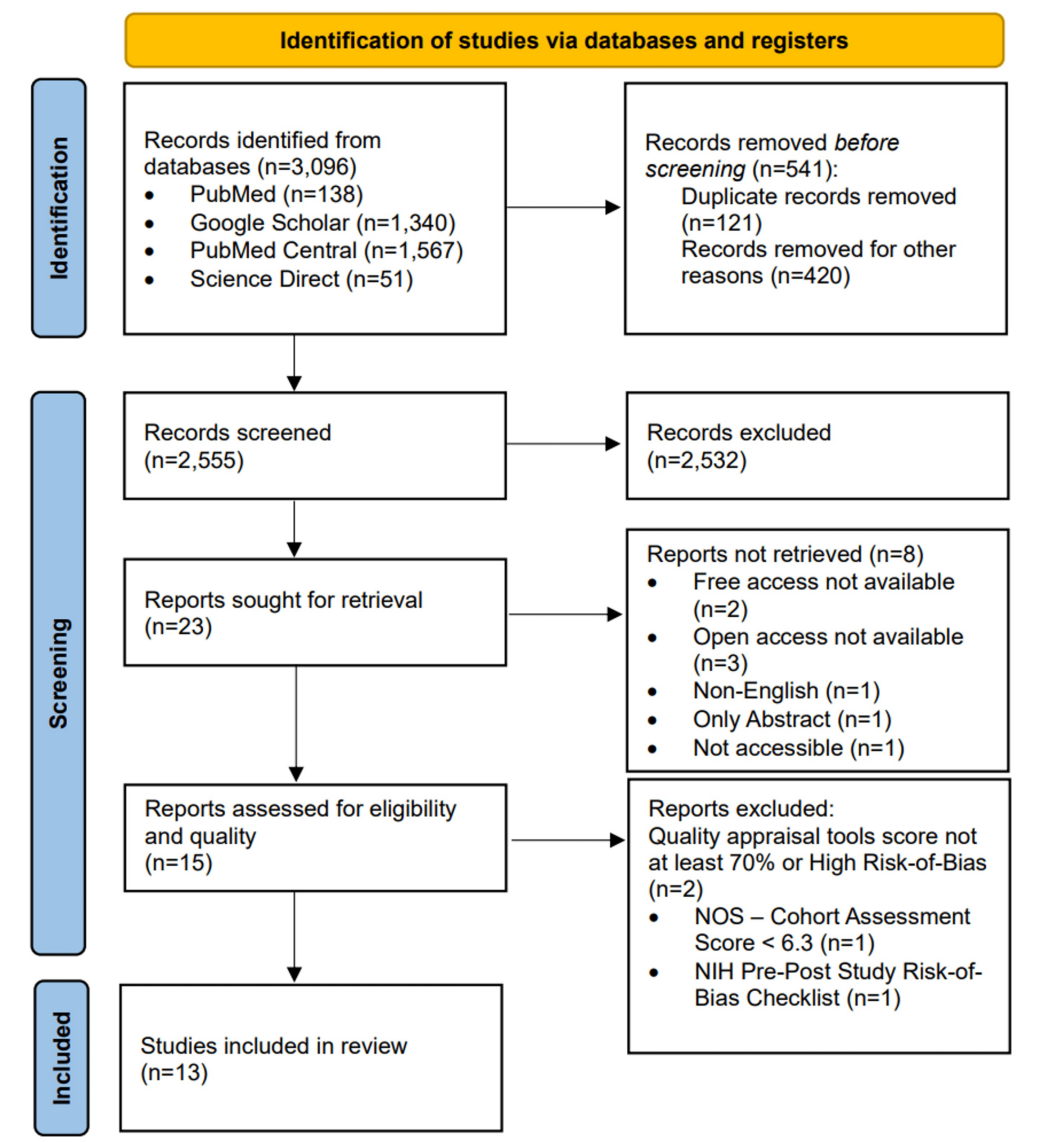
Preferred Reporting Items for Systematic Reviews and Meta-Analyses (PRISMA) flowchart NOS: Newcastle-Ottawa Scale; NIH: National Institute of Health

Using PubMed, PubMed Central, Google Scholar, and ScienceDirect databases, a total of 3096 research papers were retrieved, and 121 duplicates were removed through EndNote. From the remaining, 420 articles were discarded as they were ineligible according to the inclusion criteria. After this initial filtering, 2555 research articles remained, which were screened manually by going through the titles. Finally, 23 research articles were sought for retrieval. The 23 articles were screened by going through the abstract, full free-text, and considering the inclusion-exclusion criteria. Out of these, eight articles were not retrieved due to no free or open access, or non-English or only abstract availability. Quality assessment was performed on the final 15 papers by standardized assessment tools, and two papers were removed due to the low quality or risk of bias. Finally, 13 papers, which included case-control studies, cohort studies, systematic review and meta-analysis, and pre-post interventional studies, were considered for our study. Figure 2 shows the PRISMA 2020 flow diagram [15].

The NOS assessment tool for case-control studies was employed to evaluate seven case-control studies that met the standards, and the results are presented in Table 2. The NOS assessment tool for cohort study was used to evaluate one cohort research that met the standards, and the assessment results are presented in Table 3.

**Table 2:** Results of the Newcastle-Ottawa Scale (NOS) assessment tool for case-control studies The passing score is 7/9 Reference: [16]

Checklist Items	Abdulhadi et al. [19]	Beysel et al. [20]	Kowalczyk et al. [21]	Shatla et al. [22]	Velkoska-Nakova et al. [23]	Xu et al. [24]	Yang et al. [25]
Selection							
Is the case definition adequate?	1	1	1	1	1	1	1
Representativeness of the cases	1	1	1	1	1	1	1
Selection of controls	1	1	1	1	0	1	1
Definition of controls	1	1	1	1	1	1	1
Comparability of cohorts							
Study controls for age	1	1	1	1	0	1	1
Study controls for any additional factor(s)	1	1	1	1	0	1	1
Exposure							
Ascertainment of exposure	1	1	1	1	1	1	1
Same method of ascertainment for cases and controls	1	1	1	1	1	1	1
Non-Response rate	1	0	0	0	1	1	0
Total	9/9	8/9	8/9	8/9	6/9	9/9	8/9
Quality	Pass	Pass	Pass	Pass	Fail	Pass	Pass

**Table 3:** Results of the Newcastle-Ottawa Scale (NOS) assessment tool for cohort studies The passing score is 7/9 Reference: [16]

Checklist Items	Maiti et al. [14]
Selection	
Representativeness of the exposed cohort	1
Selection of the non-exposed cohort	1
Ascertainment of exposure	1
Demonstration that outcome of interest was not present at the start of the study	1
Comparability of cohorts	
Study controls for the most important factor (age)	1
Study controls for any additional factor(s)	1
Outcome	
Assessment of outcome	1
Was follow-up long enough for outcomes to occur?	1
Adequacy of follow-up of cohorts	1
Total	9/9
Quality	Pass

Two systematic reviews included in our study were evaluated using the AMSTAR 2 checklist, as illustrated in Table 4 [17]. Quality Assessment Tool for Before-After (Pre-Post) Studies with No Control Group by the NIH was used for pre-post interventional studies in our studies, as illustrated in Table 5 [18].

**Table 4:** Details of the Assessment of Multiple Systematic Reviews 2 (AMSTAR-2) checklist for articles retained in the study To check yes, no, can’t answer, and not applicable. The passing score is High quality (>70% Yes) Reference: [17]

Checklist Items	Kotwal et al. [26]	Chunyan et al. [27]
Did the research questions and inclusion criteria for the review include the components of Population, Intervention, Control, Outcome (PICO) framework?	Yes	Yes
Did the report of the review contain an explicit statement that the review methods were established prior to the conduct of the review and did the report justify any significant deviations from the protocol?	Yes	Yes
Did the review authors explain their selection of the study designs for inclusion in the review?	Yes	Yes
Did the review authors use a comprehensive literature search strategy?	Yes	Yes
Did the review authors perform study selection in duplicate?	Yes	Yes
Did the review authors perform data extraction in duplicate?	Yes	Yes
Did the review authors provide a list of excluded studies and justify the exclusions?	Yes	Yes
Did the review authors describe the included studies in adequate detail?	Yes	Yes
Did the review authors use a satisfactory technique for assessing the Risk of Bias (RoB) in individual studies included in the review?	Yes	Yes
Did the review authors report on the sources of funding for the studies included in the review?	Yes	Yes
If meta-analysis was performed, did the review authors use appropriate methods for statistical combination of results?	Yes	Yes
If meta-analysis was performed, did the review authors assess the potential impact of risk of bias in individual studies on the results of the meta-analysis or other evidence synthesis?	Yes	Yes
Did the review authors account for risk of bias in individual studies when interpreting/discussing the results of the review?	Yes	Yes
Did the review authors provide a satisfactory explanation for, and discussion of, any heterogeneity observed in the results of the review?	Yes	Yes
If they performed quantitative synthesis, did the review authors carry out an adequate investigation of publication bias (small study bias) and discuss its likely impact on the results of the review?	Yes	Yes
Did the review authors report any potential sources of conflict of interest, including any funding they received for conducting the review?	Yes	Yes
Quality	High	High

**Table 5:** Results of the Quality Assessment Tool for Before-After (Pre-Post) Studies with No Control Group by the National Institute of Health (NIH) To check yes, no, cannot determine, not reported, and not applicable. The passing score is High quality (>70% Yes) NA: not applicable; NR: not reported; CD: cannot determine Reference: [18]

Checklist Items	Benabdelkamel et al. [28]	Ostadrahimi et al. [29]	Raut et al. [30]	Rivera-Hernandez et al. [31]	Roohigilani et al. [32]
Was the study question or objective clearly stated?	Yes	Yes	Yes	Yes	Yes
Were eligibility/selection criteria for the study population prespecified and clearly described?	NR	Yes	No	Yes	Yes
Were the participants in the study representative of those who would be eligible for the test/service/intervention in the general or clinical population of interest?	Yes	Yes	Yes	No	Yes
Were all eligible participants that met the prespecified entry criteria enrolled?	NA	Yes	NA	CD	CD
Was the sample size sufficiently large to provide confidence in the findings?	No	No	CD	CD	CD
Was the test/service/intervention clearly described and delivered consistently across the study population?	Yes	Yes	Yes	Yes	Yes
Were the outcome measures prespecified, clearly defined, valid, reliable, and assessed consistently across all study participants?	Yes	Yes	Yes	Yes	Yes
Were the people assessing the outcomes blinded to the participants' exposures/interventions?	NA	NA	NA	NA	NA
Was the loss to follow-up after baseline 20% or less? Were those lost to follow-up accounted for in the analysis?	Yes	NR	Yes	NA	Yes
Did the statistical methods examine changes in outcome measures from before to after the intervention? Were statistical tests done that provided p values for the pre-to-post changes?	Yes	Yes	Yes	Yes	Yes
Were outcome measures of interest taken multiple times before the intervention and multiple times after the intervention (i.e., did they use an interrupted time-series design)?	NR	NR	Yes	NR	Yes
If the intervention was conducted at a group level (e.g., a whole hospital, a community, etc.) did the statistical analysis consider the use of individual-level data to determine effects at the group level?	NA	NA	NA	NA	NA
Risk of Bias	Low	Low	Low	High	Low

A total of 15 articles underwent quality appraisal, and two articles were removed following the risk-of-bias assessment. Finally, 13 papers were included in the review, and Table 6 below provides details of those included articles along with their key findings.

**Table 6:** Key findings from the papers included in the review PHT: primary hypothyroidism; LT4: levothyroxine; SCH: subclinical hypothyroidism; BMI: body mass index; LDL: low-density lipoprotein; hs-CRP: high-sensitivity C-reactive protein; TC: total cholesterol; TG: triglycerides; HDL: high-density lipoprotein; NAFLD: non-alcoholic fatty liver disease; HbA1c: hemoglobin A1C; DBP: diastolic blood pressure; ApoA: apolipoproteinA; ApoB: apolipoproteinB; Lp(a): lipoprotein(a); TSH: thyroid-stimulating hormone; FT4: free thyroxine; FBG: fasting blood glucose; HOMA: homeostatic model assessment; IR: insulin resistance; QUICKI: Quantitative Insulin Sensitivity Check Index; VLDL: low-density lipoprotein; FBS: fasting blood sugar; 2hPP: 2-hour postprandial; SBP: systolic blood pressure; QoL: quality of life; PCOS: polycystic ovary syndrome

Population Type	Reference	Study Type	Population Size	Key Findings
General	Abdulhadi et al. [19]	Case Control Study	62 PHT patients (27 newly diagnosed and 35 on LT4 therapy), 28 controls	LT4 treatment improved leptin, adiponectin, leptin-adiponectin ratio.
	Beysel et al. [20]	Case Control Study	30 SCH patients, 40 controls	LT4 treatment led to significant decrease in BMI, LDL, and hs-CRP levels; TC, TG, and HDL levels remained unchanged.
	Shatla et al. [22]	Case Control Study	325 hypothyroid patients, 325 controls	Significant reduction in NAFLD prevalence; Significant improvements in BMI, waist circumference, TC, TG, LDL, fasting blood glucose, HbA1c, and liver enzymes.
	Maiti et al. [14]	Cohort Study	60 hypothyroid patients	Significant improvement in BMI, DBP, serum insulin, fasting sugar, serum adiponectin, thyroid profile, TC, LDL, HDL, TG, subcutaneous fat; Improvement in cadiovascular risk factors.
	Kotwal et al. [26]	SR & MA	12,855 patients (hyperthyroidism + hypothyroidism cases) from 166 studies	Significant decrease in TC, LDL, HDL, TG, ApoA, ApoB, Lp(a).
	Benabdelkamel et al. [28]	Pre-Post Study	18 hypothyroid patients	LT4 treatment demonstrated notable enhancements in metabolomic profile of patients. No observed difference in TC, LDL, TG - possibly because recruited patients had lipid profiles in accepted range and limited time frame.
	Ostadrahimi et al. [29]	Pre-Post Study	105 hypothyroid patients	Significant differences in BMI, TC, TG, LDL, fasting insulin, TSH, FT4; No difference in HDL, FBG, HOMA-IR, QUICKI.
	Raut et al. [30]	Pre-Post Study	122 SCH patients	Reduction in TC, LDL, TG, VLDL; No effect on HDL, BMI.
	Roohigilani et al. [32]	Pre-Post Study	153 SCH patients	Improvement in lipid profile, insulin resistance; Significant decrease in TC, LDL, TSH levels, FBS, 2hPP, HOMA score; No effect on TG, waist circumference, BMI, uric acid, and blood pressure; But treatment helps high SBP and high DBP.
Pregnant	Xu et al. [24]	Case Control Study	164 pregnant hypothyroid patients, 407 euthyroid controls	Low FT4 levels associated with elevated blood lipid levels. Serum FT4 and lipid levels in patients could be improved by LT4 treatment.
	Yang et al. [25]	Case Control Study	20,365 pregnant patients pre-exclusion; 319 SCH patients in LT4 treatment group, 103 SCH patients in non-LT4 treatment group, 9,598 controls	Treatment improved TC and LDL; Presence of interaction effect between treatment and BMI.
Aged 60 and above	Chunyan et al. [27]	SR & MA	5000 participants from 13 studies	Significantly reduced TC, TG, LDL, ApoB; No significant increase in adverse events; No significant effect on bone mineral density, fatigue, QoL, BMI, cognitive function, depression.
Women with PCOS	Kowalczyk et al. [21]	Case Control Study	38 women with PCOS and untreated SCH, 76 women with PCOS and hypothyroidism under LT4 treatment, 76 women with PCOS and normal thyroid function	Treatment of SCH in PCOS patients does not significantly alter lipid and glucose metabolism.

Discussion

A few research projects have explored the effects of LT4 therapy on hypothyroid patients. A significant study by Raut et al. was conducted on 122 patients diagnosed with SCH and had positive thyro-peroxidase antibodies [30]. The patients recruited in the study were administered LT4 based on their TSH levels and were followed up after six to nine weeks. Significant reductions were observed in their mean TSH, TC, and LDL levels. Further supporting these findings, an observational prospective cohort study by Maiti et al. involved the recruitment of 60 hypothyroid patients to assess changes in cardiovascular risk markers, thyroid function, and other metabolic parameters [14]. The study observed significantly improved results of thyroid function tests, with reductions in TSH levels and apoA. Additionally, LT4 therapy led to notable decreases in serum lipid levels, including TC, LDL, and TG [14,30]. These findings suggest that LT4 therapy is effective in improving the lipid profiles in hypothyroid patients, and potentially reducing cardiovascular risks associated with dyslipidemia.

Beysel et al. conducted a retrospective observational study to evaluate the impact of thyroxine treatment on cardiovascular risk factors in patients with SCH [20]. The study included 30 SCH patients and 40 age- and sex-matched healthy controls. The SCH patients were treated with thyroxine 50 μg/day for three months. Before treatment, the patients had higher body mass index (BMI), serum glucose, TC, LDL, and high-sensitivity C-reactive protein (hs-CRP) levels compared to controls. After treatment, there was a significant decrease in BMI, LDL, and hs-CRP levels in the patients, while TC, TG, and HDL levels remained unchanged. The study found that TSH levels were positively correlated with BMI, glucose, LDL, and hs-CRP levels [20]. The logistic regression analysis indicated that TSH was independently associated with LDL levels.

A comprehensive systematic review and meta-analysis by Kotwal et al. reviewed 166 studies with a total of 12,855 patients, including both hyperthyroidism and hypothyroidism cases [26]. The analysis identified a statistically significant change in TC, LDL cholesterol, HDL cholesterol, and TG levels due to LT4 therapy in patients with overt hypothyroidism. However, according to the analysis, the changes in cholesterol levels were less significant in patients with SCH, and there was no statistically significant change in HDL levels [26].

An innovative study by Benabdelkamel et al. used high-resolution mass spectrometry to analyze plasma samples from 18 hypothyroid patients before and after achieving a euthyroid state with LT4 treatment [28]. Significant decreases in ceramide, phosphatidylcholine, TG, acylcarnitine, and peptides were observed post-treatment, indicating changes in fatty acid transportation and enhanced β-oxidation. Additionally, an increase in glycocholic acid suggested thyroid hormone involvement in bile acid production and secretion. Despite these findings, no notable changes in serum concentrations of TC, LDL, and TG were observed after treatment [28]. This study underscores the utility of metabolomics in understanding the molecular impact of LT4 therapy on hypothyroidism and its potential role in refining treatment approaches​​.

Shatla and Faisal investigated the relationship between overt hypothyroidism and non-alcoholic fatty liver disease (NAFLD) and assessed the impact of LT4 therapy on NAFLD [22]. Conducted on 325 hypothyroid patients and 325 controls, the study showed a significantly higher prevalence of NAFLD, elevated metabolic variables, and liver enzymes in the hypothyroidism group compared to controls. After 12 months of LT4 therapy, there was a significant reduction in NAFLD prevalence and substantial improvements in BMI, waist circumference, TC, TG, LDL, fasting blood glucose, HbA1c, and liver enzymes (alanine aminotransferase, aspartate aminotransferase, gamma-glutamyl transferase) [22].

Roohigilani et al. evaluated the impact of LT4 therapy on metabolic parameters and insulin resistance (IR) in 153 patients with SCH. After six months of LT4 treatment, there were significant improvements in TSH, T3, and T4 levels, and reductions in fasting blood sugar, fasting insulin, two-hour postprandial glucose, homeostasis model assessment estimated insulin resistance (HOMA-IR) score, TC, LDL cholesterol, and C-reactive protein (CRP) levels [32]. HDL cholesterol levels increased significantly. However, the treatment had no significant effect on TG, BMI, waist circumference, uric acid, and mean systolic and diastolic blood pressure. The number of patients with high blood pressure decreased significantly. The study concluded that LT4 therapy is beneficial for improving IR and lipid profiles in SCH patients, and it is strongly recommended for those with IR, dyslipidemia, obesity, and hypertension​​ [32].

Another study by Ostadrahimi et al. focused on the impact of LT4 on IR, lipid profiles, and BMI in hypothyroid patients [29]. Conducted on 105 untreated hypothyroid patients, the study measured insulin, fasting blood glucose, and lipid profiles at diagnosis and three months post-LT4 therapy. The findings revealed significant improvements in BMI, TC, TG, LDL, fasting insulin, and TSH levels following treatment. However, no significant changes were observed in IR as assessed by the HOMA-IR and, the quantitative insulin sensitivity check index (QUICKI) and HDL. The study concluded that while LT4 therapy significantly improves lipid profiles and BMI, it does not improve IR and suggests the need for more sensitive tests to evaluate LT4 therapy's effects on mild IR cases [29].

Another study by Abdulhadi et al. investigated the metabolic effects of hypothyroidism on leptin (LP), adiponectin (ADP), and the leptin-adiponectin ratio (LAR) [19]. The study included 62 PHT patients (27 newly diagnosed and 35 on LT4 therapy) and 28 healthy controls, and their anthropometric, lipid, and pressure profiles were evaluated, along with TSH, T3, T4, LP, and ADP serum levels. The study concluded that hypothyroidism is associated with poor cardio-metabolic profiles and high LAR, with LT4 therapy improving all the evaluated parameters [19].

Table 7 presents the compiled findings from the reviewed research papers, illustrating the changes in lipid profiles of hypothyroid patients before and after LT4 treatment.

**Table 7:** Changes in the lipid profile of hypothyroid patients before and after levothyroxine (LT4) therapy ↑ (upward arrow): increase in the parameter; ↓ (downward arrow): decrease in the parameter TSH: thyroid-stimulating hormone; FT4: free thyroxine; T3: triiodothyronine; TC: total cholesterol; LDL: low-density lipoprotein; TG: triglycerides; HDL: high-density lipoprotein; LT4: levothyroxine References: [14,19,20,22,26,28-30,32]

Hypothyroidism Patients	TSH	FT4	T3	TC	LDL	TG	HDL
Before LT4 therapy	↑	↓	↓	↑	↑	↑	↓
After LT4 therapy	↓	↑	↑	↓	↓	No change or ↓	No change or ↑

Effect of LT4 Therapy on Pregnant Patients

The effects of LT4 therapy on lipid profiles and overall health outcomes during pregnancy have been a subject of significant interest. Xu et al. conducted a retrospective study involving 164 pregnant patients with isolated hypothyroxinemia and 407 euthyroid controls [24]. The study investigated the association between low FT4 levels and lipid metabolism in pregnant women, as well as the impact of LT4 treatment on these parameters and found that hypothyroid patients had significantly higher levels of TC, TG, LDL cholesterol, and apoB when compared to controls. An inverse correlation between FT4 and TG levels was observed, which remained significant after adjusting for pre-pregnancy BMI. Patients receiving LT4 treatment showed a slower progression of hypercholesterolemia during pregnancy compared to those receiving dietary iodine supplementation [24].

Yang et al. conducted a large-scale cohort study involving 20,365 pregnant patients, including 319 in the subclinical hypothyroidism LT4 treatment group, 103 in the SCH non-intervention group, and 9598 controls [25]. The research found that serum lipid levels, specifically TC, TG, LDL, and HDL, vary significantly throughout pregnancy and established reference ranges for these lipids in the first and third trimesters. The study concluded that LT4 treatment effectively reduced TC and LDL levels in pregnant women with SCH, with treatment effects influenced by BMI. Notably, the treatment only lowered HDL levels in obese women, exacerbating the negative effects of obesity [25].

The findings underscore the clinical importance of routine thyroid function screening and managing lipid levels in pregnant women with SCH, especially those with high pre-pregnancy BMI or hyperlipidemia, to potentially prevent adverse pregnancy outcomes such as gestational diabetes mellitus (GDM), preeclampsia, and preterm delivery [24,25].

Effect of LT4 Therapy on Older Patients

A systematic review and meta-analysis by Zhao et al. examined the effects of LT4 treatment on older patients with subclinical hypothyroidism [27]. Through a comprehensive meta-analysis of 13 studies, including approximately 5000 participants aged 60 and above, the research revealed that LT4 significantly reduced TC, TG, LDL cholesterol, and apoB levels, suggesting a potential benefit in lipid profile improvement. However, LT4 did not considerably affect bone mineral density, fatigue, hypothyroid symptoms, quality of life, BMI, cognitive function, depression, blood pressure, serum creatinine, fasting blood glucose, HDL, or apoA. The study concluded that while LT4 can improve lipid profiles, its use should be cautious in older patients to avoid unnecessary and excessive treatment [27].

Effect of LT4 Therapy on Polycystic Ovary Syndrome (PCOS) Patients

Investigating the relationship between SCH, PCOS, and the effects of LT4 treatment, Kowalczyk et al. studied 190 women with PCOS by dividing them into three groups: PCOS with untreated SCH, PCOS with SCH under LT4 treatment, and PCOS with normal thyroid function [21]. There were no significant differences in lipid profile and glucose metabolism among the groups, and BMI was identified as the primary factor affecting serum lipids, fasting glucose, fasting insulin, and insulin resistance. TSH levels were associated with TC and LDL cholesterol levels, while BMI significantly influenced HDL cholesterol, TG, glucose, and IR. The study concluded that SCH causes mild lipid serum alterations in women with PCOS, but BMI has a more dominant impact on glucose and insulin metabolism. Treatment of SCH in PCOS does not significantly alter lipid and glucose metabolism; therefore, it is important to manage the BMI of these patients [21].

Limitations

The review has taken into consideration research articles published after 2020 by searching only four databases and has included only studies published in the English language. This may have omitted relevant studies published before 2020, not indexed in the selected databases, or published in other languages. In addition, only full free-text articles were acquired, and thus, this may have introduced a selection bias, potentially limiting the comprehensiveness of the systematic review.

## Conclusions

Hypothyroidism is a thyroid gland disorder characterized by low or nil production of thyroid hormones. Since the thyroid hormone influences lipid metabolism, a derangement is seen in the lipid profile of hypothyroid patients, resulting in elevated TC, LDL, and TG values. When such hypothyroid patients are treated with LT4 therapy, a significant decrease in TC, LDL, and TG values is seen. This highlights the importance of starting timely LT4 therapy in hypothyroid patients to maintain normal lipid levels and reduce associated cardiovascular risk.
